# Supplementary material for: Phase transformation mechanism in lithium manganese nickel oxide revealed by single-crystal hard X-ray microscopy
Source: Nat Commun. 2017 Feb 1;8:14309. doi: 10.1038/ncomms14309 (PMC5296648; doi:10.1038/ncomms14309)
Supplement: Supplementary Information — Supplementary Figures 1-3 [file ncomms14309-s1.pdf]

Supporting information

a

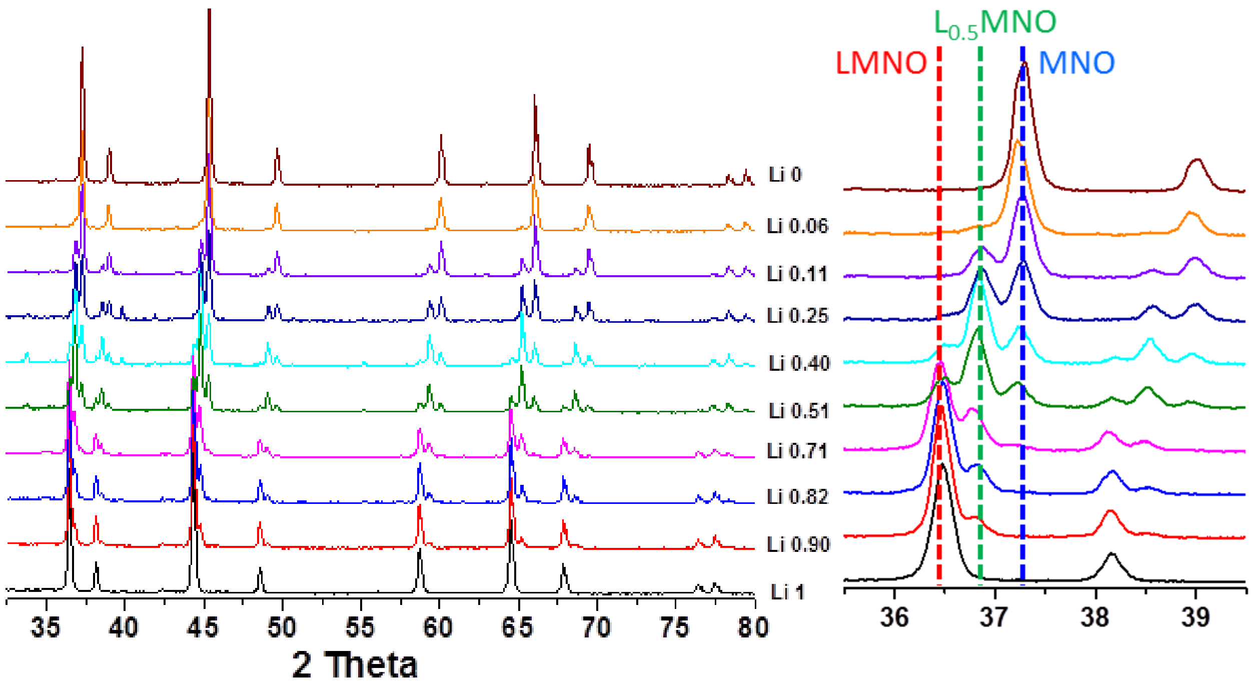

b

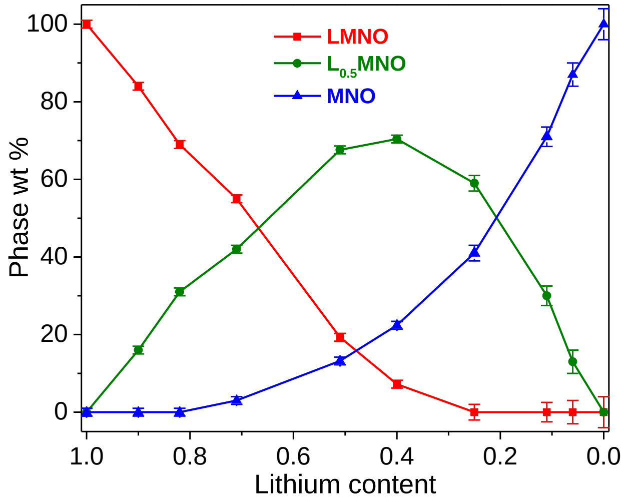

Supplementary Figure 1. **X-ray diffraction (XRD) measurement of the samples**. a) Room-temperature XRD patterns and b) phase composition of the as-prepared $Li_xMn_{1.5}Ni_{0.5}O_4$ crystal samples.

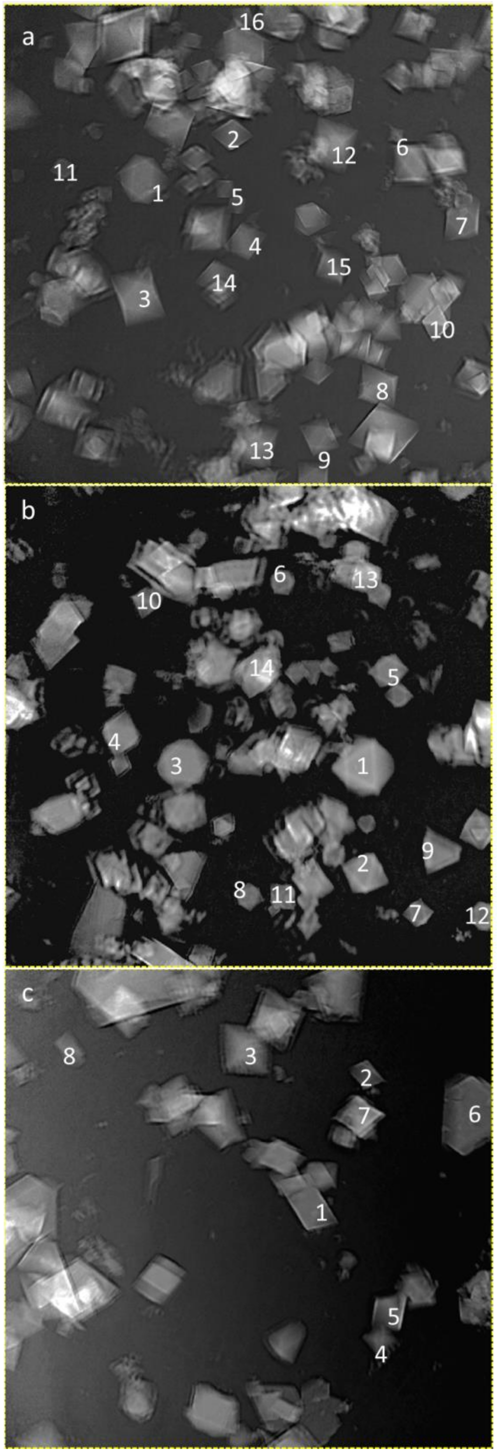

Supplementary Figure 2. **TXM FOVs of the $Li_xMn_{1.5}Ni_{0.5}O$ crystal samples**. a) x=0.82, b) x=0.71 and c) x=0.51. Each image corresponds to a 25 µm by 25 µm area. The numbers indicate the microcrystals selected for analysis.

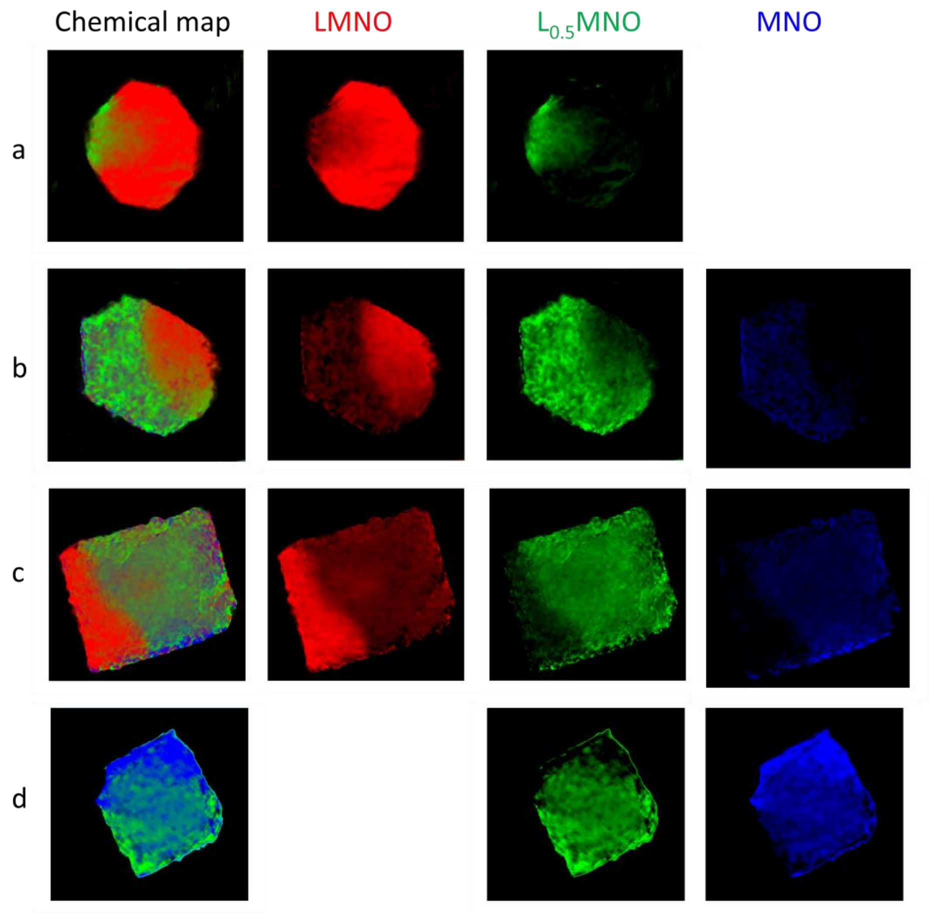

Supplementary Figure 3. **Chemical mapping along with the individual phase maps of the particles shown in Figure 2**. a) $x = 0.82$, b) $x = 0.71$, c) $x = 0.51$ and d) $x = 0.25$.
